# Graphitic Carbon Nitride-Decorated Cobalt Diselenide Composites for Highly Efficient Hydrogen Evolution Reaction

**DOI:** 10.3390/ijms262412188

**Published:** 2025-12-18

**Authors:** Abu Talha Aqueel Ahmed, Saravanan Sekar, Sutha Sadhasivam, Balaji Murugan, Sangeun Cho, Youngmin Lee, Sejoon Lee, Sankar Sekar

**Affiliations:** 1 Division of System Semiconductor, Dongguk University-Seoul, Seoul 04620, Republic of Korea; abutalha.aa@dongguk.edu (A.T.A.A.); ymlee@dongguk.edu (Y.L.); sejoon@dongguk.edu (S.L.); 2 Department of Mechanical Engineering, K. Ramakrishnan College of Technology, Trichy 621112, Tamil Nadu, India; nanosaran007@gmail.com; 3 Department of Chemistry, CMS College of Engineering, Namakkal 637003, Tamil Nadu, India; suthaasridhar@gmail.com; 4 Department of Electrical & Computer Engineering, National University of Singapore, Singapore 117608, Singapore; balajim@nus.edu.sg; 5 Quantum-Functional Semiconductor Research Center, Dongguk University-Seoul, Seoul 04620, Republic of Korea

**Keywords:** cobalt diselenide, graphitic carbon nitride, electrocatalysts, nanoparticles, hydrogen evolution reaction

## Abstract

Transition-metal dichalcogenides have emerged as promising non-noble-metal electrocatalysts for efficient hydrogen production through the hydrogen evolution reaction (HER). In this work, we fabricated the graphitic carbon nitride-decorated cobalt diselenide (gC_3_N_4_-CoSe_2_) nanocomposites via the facile hydrothermal method. The prepared gC_3_N_4_-CoSe_2_ nanocomposites displayed an interconnected and aggregated morphology of gC_3_N_4_-decorated CoSe_2_ nanoparticles with offering large surface area of 82 m^2^/g. The gC_3_N_4_-CoSe_2_ nanocomposites exhibited excellent HER activity with a low overpotential (141 mV) and tiny Tafel slope (62 mV/dec) with excellent durability for 100 h at 10 mA/cm^2^ in an alkaline electrolyte. These outstanding HER performances of gC_3_N_4_-CoSe_2_ can be ascribed to the synergistic interaction between the electrochemically active porous CoSe_2_ nanoparticles and the highly conductive gC_3_N_4_ nanosheets. These results indicate that the gC_3_N_4_-CoSe_2_ nanocomposites hold promising and efficient HER electrocatalysts for sustainable green hydrogen production.

## 1. Introduction

The increasing global energy demand, depletion of conventional fossil fuels, and escalating environmental deterioration have intensified the pursuit of sustainable, clean, and renewable energy alternatives. Recently, hydrogen has emerged as a highly promising alternative energy carrier due to its environmental sustainability, extraordinary energy density, and abundant availability [1,2,3]. Among the various hydrogen fabrication methods, water electrolysis is a prospective and efficient method for hydrogen production via hydrogen evolution reaction (HER) because of its low energy consumption and zero carbon emissions [4,5]. Although platinum (Pt)-based derivates demonstrate excellent HER performance, owing to their poor sluggish kinetics, inferior durability, and expensiveness their practical applications were limited [6,7,8]. Consequently, the development of advanced HER catalysts that exhibit high activity, outstanding stability, and cost-effectiveness remains a crucial challenge in achieving sustainable hydrogen energy technologies [9,10].

Recently, enormous efforts have been devoted to developing alternative catalysts (e.g., chalcogenides, sulfides, hydroxides, phosphides, oxides, and carbides) that are highly earth-abundant, cost-effectiveness, environmentally sustainable, and possess high electrochemical activities [11,12,13,14]. Owing to their tunable structures, low cost, intrinsic activity, and unique physicochemical properties, transition metal dichalcogenides (TMDs)-based materials have recently attracted considerable attention as promising electrocatalysts for HER [15,16,17]. Among various TMDs, cobalt diselenide (CoSe_2_) has emerged as a highly promising electrocatalysts for HER due to its tunable electronic structure, high chemical stability, low cost, huge availability, and good intrinsic activity [18,19,20,21]. However, the pure CoSe_2_ nanostructures demonstrate limited HER activity owing to their poor electrical conductivity, insufficient active sites, sluggish reaction kinetics, and agglomerate during the catalytic process [22,23,24]. Therefore, several approaches have been developed to hybridize CoSe_2_ nanostructures with transition metal oxides/chalcogenides, and carbon-based materials to enhance their catalytic HER efficiency and stability [25,26,27,28,29]. Recently, graphitic carbon nitride (gC_3_N_4_) has gained considerable attention as a promising co-catalyst owing to its outstanding chemical stability, facile synthesis, tunable electronic structure, low cost, earth abundance, distinctive 2D layered structure, and easily adjustable framework [30,31,32,33]. The integrating gC_3_N_4_ with CoSe_2_ can effectively enhance electrical conductivity, increase the surface area, and expose additional active sites, thereby substantially improving the electrocatalytic HER performance [27,28,34]. For instance, Dileepkumar et al. [27] prepared CoSe_2_ grafted onto g-C_3_N_4_ via a facile hydrothermal process and achieved an overpotential of 210 mV at −50 mA/cm^2^ for HER in an alkaline electrolyte. Sekar et al. [33] prepared 2D–2D gC_3_N_4_–MoS_2_ nanocomposites via the sonication method and demonstrated the overpotential of 156 mV at −10 mA/cm^2^ for HER in 1 M KOH. Priyakshree et al. [35] synthesized CoS_2_/g-C_3_N_4_ using the hydrothermal technique and showed the excellent HER performance of 230 mV at −10 mA/cm^2^ in 1 M KOH. Zulqarnain et al. [28] synthesized CoSe_2_ embedded in g-C_3_N_4_ using hydrothermal method and exhibited the overpotential of 193 mV at −20 mA/cm^2^ for HER in an acidic electrolyte. Dileepkumar et al. [36] prepared the NiSe_2_ on S-doped g-C_3_N_4_ composites by using facile hydrothermal process and achieved the overpotential of 128 mV at −10 mA/cm^2^ in 1 M KOH. Krishnamurthy et al. [37] fabricated sea urchin-like Ni-doped CoSe_2_/g-C_3_N_4_ using solvothermal method and achieved the excellent electrocatalytic performances. Despite all of the above, the electrocatalytic HER performance of the g-C_3_N_4_-CoSe_2_ nanocomposites has rarely been investigated.

In spite of all the above, we synthesized the gC_3_N_4_-CoSe_2_ nanocomposites via the facile hydrothermal method and examined their electrocatalytic HER performances. The gC_3_N_4_-CoSe_2_ nanocomposites showed excellent HER performance with a low overpotential of 141 mV at 10 mA/cm^2^ in 1 M KOH. Herein, the material synthesis, material characteristics, and electrocatalytic HER activity of the gC_3_N_4_-CoSe_2_ nanocomposites are technically evaluated and deliberated in detail.

## 2. Results and Discussion

**Figure 1:**
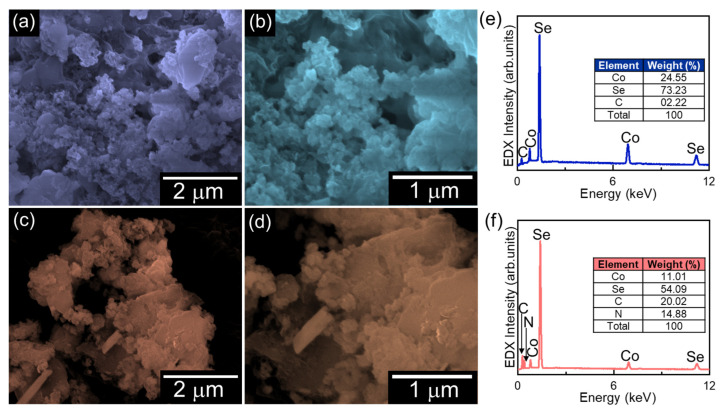
Low and high magnification FE-SEM images of (**a**,**b**) CoSe_2_ nanoparticles and (**c**,**d**) gC_3_N_4_-CoSe_2_ nanocomposites. EDX spectra of (**e**) CoSe_2_ and (**f**) gC_3_N_4_-CoSe_2_.

The microstructure of the bare CoSe_2_ and gC_3_N_4_-CoSe_2_ nanocomposites were examined using FE-SEM, as presented in Figure 1a–d. The bare CoSe_2_ sample exhibits interconnected and irregularly aggregated porous nanoparticles (Figure 1a,b). In contrast, the gC_3_N_4_-CoSe_2_ nanocomposites clearly revealed the gC_3_N_4_ nanosheets anchored on the CoSe_2_ nanoparticles, forming a porous and densely hybridized structure (Figure 1c,d). The in situ EDX spectra (Figure 1e,f) confirm the existence of Co, Se, C, and N elements in both samples, indicating their high purity and absence of any detectable impurities.

**Figure 2:**
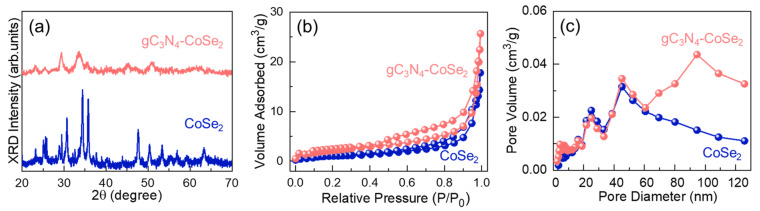
(**a**) XRD patterns, (**b**) nitrogen adsorption–desorption isotherm, and (**c**) pore characteristics of the CoSe_2_ nanoparticles and gC_3_N_4_-CoSe_2_ nanocomposites.

The crystallographic characteristics of the bare CoSe_2_ nanoparticles and gC_3_N_4_-CoSe_2_ nanocomposites were characterized by Powder XRD measurements. Figure 2a demonstrates the XRD pattern of the bare CoSe_2_ and gC_3_N_4_-CoSe_2_. Both samples displays distinct diffraction peaks at 23.65, 29.04, 30.67, 34.42, 35.83, 43.81, 47.75, 50.37, 53.37, 55.44, 56.94, 59.29, and 63.21° corresponding to the (110), (011), (101), (111), (120), (121), (211), (002), (031), (221), (131), (310), and (122) planes of orthorhombic CoSe_2_ (JCPDS card no: 53-0449), respectively [38,39,40,41,42]. Moreover, no distinct gC_3_N_4_ peak was observed in the gC_3_N_4_-CoSe_2_ nanocomposite because of the low amount of gC_3_N_4_ and high diffraction intensity of crystalline CoSe_2_ nanoparticles in the composite system. The reduced peak intensity further confirms the successful incorporation of gC_3_N_4_ onto the CoSe_2_ surface, indicating the hybrid composite formation [27,43].

The BET and BJH techniques were employed to investigate the specific surface area and porosity characteristics of the bare CoSe_2_ nanoparticles and gC_3_N_4_-CoSe_2_ nanocomposites. Figure 2b nitrogen adsorption–desorption isotherm curves of the bare CoSe_2_ nanoparticles and gC_3_N_4_-CoSe_2_ nanocomposites. Both samples showed a Type IV adsorption curve with a typical H3 hysteresis pattern (categorized by IUPAC), indicating the mesoporous nature of the materials [20,27,33,44]. From BET analysis, the specific BET surface areas of the bare CoSe_2_ nanoparticles and gC_3_N_4_-CoSe_2_ nanocomposites are 35 and 82 m^2^/g, respectively. The gC_3_N_4_-CoSe_2_ nanocomposites exhibited a high specific surface area compared to the bare CoSe_2_ nanoparticles and other reported catalysts, as summarized in Table S1. In addition, the BJH analysis revealed pore surface areas of 21 m^2^/g for bare CoSe_2_ nanoparticles and 67 m^2^/g for the gC_3_N_4_-CoSe_2_ nanocomposite (Figure 2c). Furthermore, the average pore diameters of the bare CoSe_2_ nanoparticles and gC_3_N_4_-CoSe_2_ nanocomposites were 28.57 and 17.16 nm, respectively. The gC_3_N_4_-CoSe_2_ nanocomposite also showed a higher total pore volume (0.0335 cm^3^/g) compared to the bare CoSe_2_ nanoparticles (0.0237 cm^3^/g), attributed to the incorporation of gC_3_N_4_ nanosheets into the composite system. The enhanced surface area and porous structure of the gC_3_N_4_-CoSe_2_ nanocomposite facilitate faster ion transport and greater accessibility of active sites, significantly enhancing HER performance in alkaline media.

**Figure 3:**
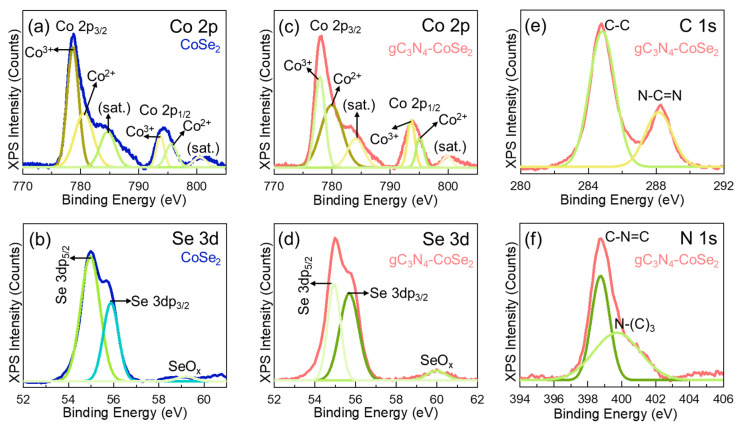
(**a**) Co 2p and (**b**) Se 3d of the XPS core levels spectra of CoSe_2_ nanoparticles. (**c**) Co 2p, (**d**) Se 3d, (**e**) C 1s, and (**f**) N 1s of the XPS core level spectra of gC_3_N_4_-CoSe_2_ nanocomposites.

The surface chemical composition and ionic states of the bare CoSe_2_ nanoparticles, pristine gC_3_N_4_ nanosheets and gC_3_N_4_-CoSe_2_ nanocomposites were characterized by XPS measurement. The XPS full survey spectra of the bare CoSe_2_ nanoparticles, pristine gC_3_N_4_ nanosheets and gC_3_N_4_-CoSe_2_ nanocomposites clearly confirmed the presence of Co, Se, N and C components (Figure S1a–c). For the Co 2p core level spectra (Figure 3a), the bare CoSe_2_ nanoparticles exhibited two major binding energy at 778.7 and 794.3 eV are ascribed to the Co 2p_3/2_ and Co 2p_1/2_, respectively, among their corresponding satellites peaks [26]. After deconvolution, the distinct binding energy at 778.9 and 793.8 eV are accredited to the Co^3+^ 2p_3/2_ and Co^3+^ 2p_1/2_, indicating the existence of Co-Se bonds [43,45]. Meanwhile, the peaks at 780.5 and 795.7 eV correspond to Co^2+^ 2p_3/2_ and Co^2+^ 2p_1/2_ associated with Co-O bonds formed through slight surface oxidation [46,47]. This clearly indicates the presence of both Co^2+^ and Co^3+^ oxidation states in the CoSe_2_ nanoparticles. For Se 3d (Figure 3b), the two binding energy at 54.9 and 55.8 eV are relating to the Se 3d_5/2_ and Se 3d_3/2_, respectively [38]. Additionally, the broad peak observed at 60.0 eV correspond to the SeO_x_ due to the surface oxidation of Se [40,48]. The C 1s spectrum of pristine gC_3_N_4_ nanosheets (Figure S2a) showed two distinct peaks at 284.9 and 287.9 eV are ascribed to the C-C and N-C=N bonds, respectively [27,49]. Similarly, the N 1s spectrum of pristine gC_3_N_4_ nanosheets (Figure S2b) obtained two peaks at 398.9 eV and 399.8 eV, accrediting to the pyridinic C–N=C and triazine N–(C_3_) bonds, respectively [33,50]. The gC_3_N_4_-CoSe_2_ nanocomposites exhibited characteristic features similar to those of bare CoSe_2_ nanoparticles and pristine gC_3_N_4_ nanosheets (Figure 3c–f). However, compared to the individual components, the binding energies in the composite shifted toward lower values, indicating effective electronic interaction between CoSe_2_ and gC_3_N_4_ [36,51]. Moreover, the reduced N–C=N intensity and the increased C–C contribution in the C 1s spectrum suggest that the interaction with CoSe_2_ partially modifies the heptazine framework of gC_3_N_4_, leading to the development of carbon-rich regions within the structure [49,50,52]. These results clearly demonstrate strong electronic coupling between CoSe_2_ and gC_3_N_4_ in the composite system, which is beneficial for improving HER performance.

**Figure 4:**
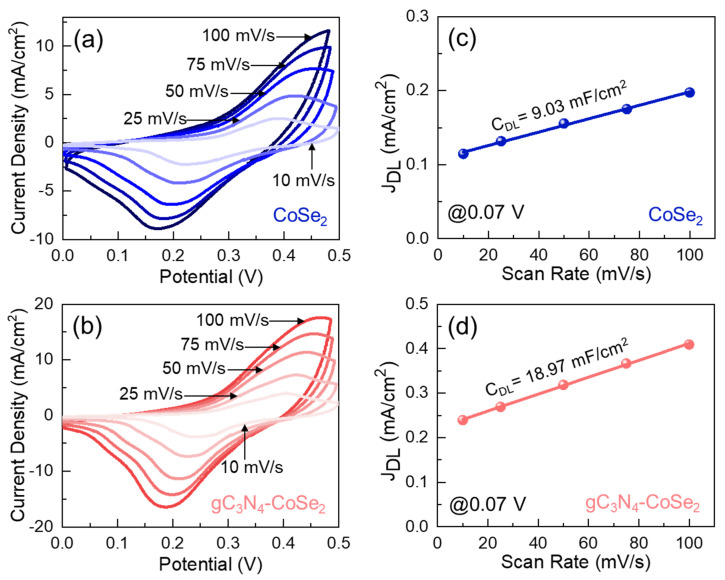
CV curves of the (**a**) CoSe_2_ and (**b**) gC_3_N_4_-CoSe_2_ catalysts. Non-Faradaic double-layer charging current (*J*_DL_) at 0.07 V as a function of scan rate for (**c**) CoSe_2_ and (**d**) gC_3_N_4_-CoSe_2_ catalysts.

The HER performance of the bare CoSe_2_ nanoparticles and gC_3_N_4_-CoSe_2_ nanocomposites was examined using CV measurements. As shown in Figure 4a,b, both catalysts exposed distinct oxidation and reduction signals, indicating typical pseudocapacitive activities originating arising from faradaic redox reactions. With increasing scan rate from 10 to 100 mV/s, the current density also increased, suggesting efficient ion transport and low diffusion resistance. Compared with bare CoSe_2_, the gC_3_N_4_-CoSe_2_ composite showed a larger CV area and higher current response, suggesting more accessible active sites and enhanced electrical conductivity. The gradual increase in redox peak intensity with increasing scan rate further indicated that the redox process mainly occurred on the catalyst surface, demonstrating the excellent electrochemical stability and improved catalytic performance of the gC_3_N_4_-CoSe_2_ composite. To further clarify the enhanced electrocatalytic behavior of the gC_3_N_4_-CoSe_2_ catalyst, the electrochemically active surface area (ECSA) was evaluated using Equations (1) and (2). The ECSA values were derived from the non-faradaic CV regions (0–0.1 V) for both CoSe_2_ and gC_3_N_4_-CoSe_2_ at 0.07 V (Figure S3a,b). From Figure 4c,d, the C_DL_ values were determined to be 9.03 and 18.97 mF/cm^2^ for CoSe_2_ and gC_3_N_4_-CoSe_2_, respectively. Using these CDL values in Equation (2), the ECSA values were 225 cm^2^ for CoSe_2_ and 474 cm^2^ for gC_3_N_4_-CoSe_2_. The higher ECSA of gC_3_N_4_-CoSe_2_ indicated that gC_3_N_4_ incorporation increased the number of active sites and enhanced the catalytic activity of the composites.

**Figure 5:**
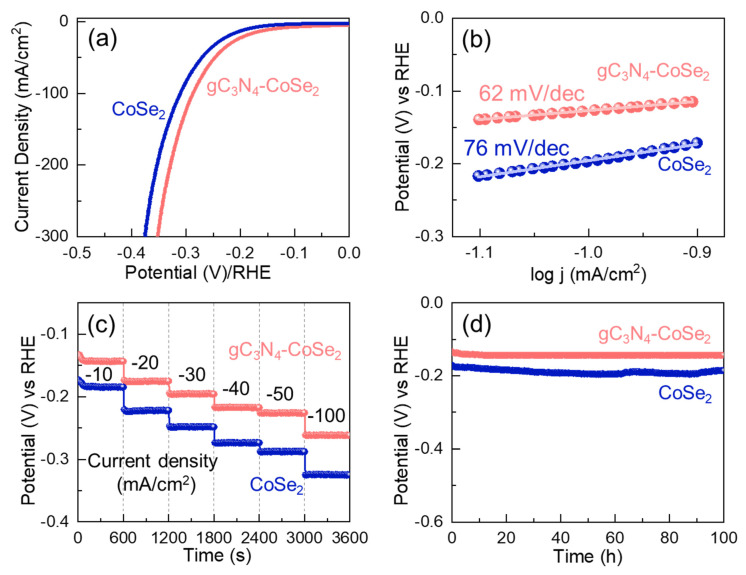
HER performances of the CoSe_2_ and gC_3_N_4_-CoSe_2_ catalysts. (**a**) *i_R_*-corrected LSV curves, (**b**) Tafel plots, (**c**) chronopotentiometric profiles at different current densities (−10 to −100 mA/cm^2^), and (**d**) long-term stability characteristics.

The LSV polarization curve provides valuable insight into the intrinsic electrocatalytic activity of the materials toward the HER process. Figure 5a displays the *i_R_*-corrected LSV polarization curves for CoSe_2_ and gC_3_N_4_-CoSe_2_ performed at 1 mV/s. Based on the LSV data and using Equations (3) and (4), the overpotential (η) of CoSe_2_ and gC_3_N_4_-CoSe_2_ were measured to be 187 and 141 mV at −10 mA/cm^2^, respectively. Compared with bare CoSe_2_, the gC_3_N_4_-CoSe_2_ nanocomposite showed a lower η due to its larger ECSA and higher number of active sites. Furthermore, the gC_3_N_4_-CoSe_2_ catalyst showed a comparable or even lower η value than those reported in the literature (Table 1), proving its superior catalytic efficiency. Moreover, the improved HER kinetic were evaluated via the Tafel slope calculated from Equation (5). As illustrated in Figure 5b, the *S*_T_ values for CoSe_2_ and gC_3_N_4_-CoSe_2_ were calculated to be 76 and 62 mV/dec, respectively. The attained *S*_T_ values suggests that the CoSe_2_ and gC_3_N_4_-CoSe_2_ catalysts arises the Volmer–Heyrovsky mechanism, where the electrochemical desorption step ($H_{\mathrm{ads}}+{\;H}_{2}O+{\;e}^{-}\;\to {\;H}_{2}+{\mathrm{OH}}^{-}$) served as the rate-determining stage. The gC_3_N_4_-CoSe_2_ catalyst exhibited a smaller Tafel slope than the bare CoSe_2_ and those reported in previous studies (Table 1). Compared to other electrocatalysts, the gC_3_N_4_-CoSe_2_ catalyst showed superior HER activity with lower *η* and *S*_T_ values, owing to its larger ECSA, higher porosity, and high electrical conductivity with enhanced intrinsic reaction kinetics. Specifically, the increase in electrochemically active sites combined with the improved electrical conductivity leads to the faster reaction kinetics. This interpretation is further supported by the ECSA-corrected LSV curve analysis (see Figure S4).

To further evaluate the catalytic stability under different operating conditions, multi-step chronopotentiometry (CP) tests were characterized at progressively increasing current densities ranging from −10 to −100 mA/cm^2^, with each step maintained for 10 min. Figure 5c shows the multi-step CP slopes of the CoSe_2_ and gC_3_N_4_-CoSe_2_ catalysts. Both CoSe_2_ and gC_3_N_4_-CoSe_2_ catalysts exhibited stable potential responses with rapid recovery at each step, indicating excellent reversibility and strong catalytic durability. However, the bare CoSe_2_ catalyst showed higher potential at all current densities, suggesting lower charge-transfer efficiency and slower hydrogen evolution kinetics. In contrast, the gC_3_N_4_-CoSe_2_ catalyst demonstrated superior performance, attributed to its highly conductive hybrid composite system that enabled efficient ion diffusion, faster electron transport, and reduced interfacial resistance at the electrode–electrolyte interface. The long-term durability of the catalysts was further assessed through continuous CP stability test at a constant current density of −10 mA/cm^2^ for 100 h, as shown in Figure 5d. The gC_3_N_4_-CoSe_2_ catalyst exhibited excellent operational stability with minimal potential degradation, while the CoSe_2_ catalyst showed a gradual increase in overpotential overtime. The superior durability of the gC_3_N_4_-CoSe_2_ catalyst could be attributed to the strong interfacial coupling between CoSe_2_ and gC_3_N_4_ within the composite system, which reduced structural degradation and prevented active site detachment during prolonged electrolysis, as supported by the post-stability LSV curves (Figure S5). These results indicated that the gC_3_N_4_-CoSe_2_ catalyst acted as a highly efficient and durable electrocatalyst for hydrogen evolution. After the HER stability test, we performed FE-SEM measurements to observe the changes in microstructural characteristics of the catalysts. From FE-SEM measurements, the CoSe_2_ catalyst displayed the aggregated structure of the nanoparticles (see Figure S6a). However, the gC_3_N_4_-CoSe_2_ catalyst still retained its original structure of the gC_3_N_4_ nanosheets-decorated CoSe_2_ nanocomposites (see Figure S6b).

**Figure 6:**
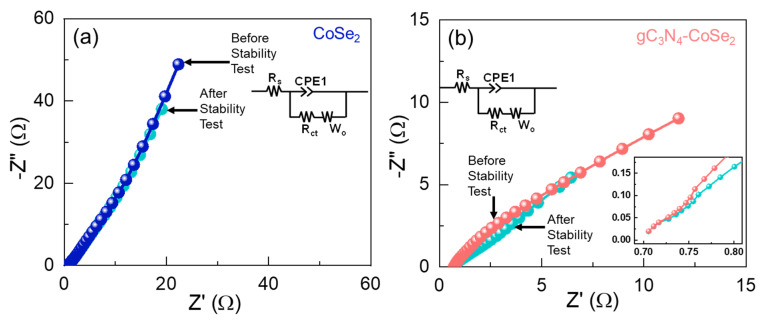
Nyquist plots of (**a**) CoSe_2_ and (**b**) gC_3_N_4_-CoSe_2_ catalysts before and after the stability test (zoomed image of (**b**)). The insets show the corresponding equivalent circuit models of the working catalysts.

Finally, to further elucidate the improved HER kinetics and resistive characteristics of the CoSe_2_ and gC_3_N_4_-CoSe_2_ catalysts were analyzed by EIS measurement. Figure 6 indicates the Nyquist plots of CoSe_2_ and gC_3_N_4_-CoSe_2_ with their corresponding equivalent circuit (inset). The EIS curves reveal a distinct difference in the quasi-parabolic curves between the CoSe_2_ and gC_3_N_4_-CoSe_2_ catalysts. The low-frequency response was attributed to the dispersion of electrolytes over the catalyst surface [22,40,63], while the high-frequency region was associated with the series resistance (R_s_) and charge-transfer resistance (R_ct_) of the catalysts [28,48,64]. From the fitted EIS curves using the equivalent circuit model (insets of Figure 6a,b), the R_s_ and R_ct_ values were measured to be 0.75 and 8.61 Ω for CoSe_2_ and 0.70 and 0.13 Ω for gC_3_N_4_-CoSe_2_. The lower resistance of the gC_3_N_4_-CoSe_2_ catalyst indicated improved charge-transfer efficiency compared to bare CoSe_2_, which can be attributed to the enhanced electrical conductivity and higher porosity provided by the incorporated gC_3_N_4_ nanosheets. The gC_3_N_4_-CoSe_2_ electrode exhibits a significantly smaller quasi-parabolic curve and the steeper Warburg impedance compared to bare CoSe_2_ catalyst, indicating a comparatively lower charge-transfer resistance and improved efficient ion diffusion, which is a result of the facilitated charge transport across the electrode–electrolyte interface. The improvement can be ascribed to the strong interfacial coupling between the conductive and highly porous gC_3_N_4_ nanosheets and CoSe_2_ nanoparticles, which facilitates rapid charge migration across the heterointerface and minimizes recombination losses. After long-term stability test, the EIS analysis revealed an almost insignificant change in the charge-transfer resistance (see inset Figure 6b), confirming the excellent durability of the catalyst due to surface reformation and strengthened electrode–electrolyte interactions. These results revealed the remarkable promise of the hydrothermally synthesized gC_3_N_4_-CoSe_2_ nanocomposites as efficient and durable HER electrocatalysts for sustainable green hydrogen production.

## 3. Materials and Methods

### 3.1. Materials

All the regents including cobalt acetate tetrahydrate (Co(CH_3_COO)_2_·4H_2_O, ≥98.0%), ethylenediamine (NH_2_CH_2_CH_2_NH_2_, ≥99%), sodium selenite (Na_2_SeO_3_, 99%), carbohydrazide (CO(NHNH_2_)_2_, 98%), potassium hydroxide (KOH, ≥85%), melamine (C_3_H_6_N_6_, 99%) were procured from Sigma-Aldrich (Seoul, Republic of Korea) and used as received without additional purification. Deionized (DI) water was utilized throughout the experiments to prevent any form of contamination.

### 3.2. Synthesis of CoSe_2_ Nanoparticles

**Figure 7:**
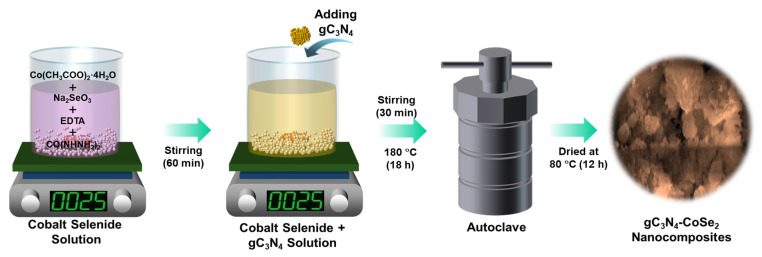
Schematic illustration of the hydrothermal synthesis of CoSe_2_ nanoparticles and gC_3_N_4_-CoSe_2_ nanocomposites.

Figure 7 illustrates the schematic diagram of the hydrothermal process used to synthesize the gC_3_N_4_-CoSe_2_ nanocomposites. The orthorhombic CoSe_2_ nanoparticles were synthesized using a simple hydrothermal approach. Initially, 0.5 g of Co(CH_3_COO)_2_·4H_2_O, 0.8 g of Na_2_SeO_3_ and 6 mL of ethylenediamine were dissolve in 50 mL of DI water and stirred for 10 min. Then, 40 mmol of carbohydrazide was slowly added dropwise to the solution and stirred for another 60 min. The above solution was moved to the 100 mL of Teflon-lined autoclave and maintained at 180 °C for 18 h. After the reaction mixture was cooled to room temperature (RT), the precipitate was amassed, washed five times with DI water, and dried at 80 °C for 12 h to achieve pure CoSe_2_ nanoparticles.

### 3.3. Synthesis of gC_3_N_4_ Nanosheets

The gC_3_N_4_ nanosheets were prepared from melamine using a simple pyrolysis approach, as described in our previous work [33]. Primarily, 5 g of melamine was placed in a covered alumina crucible and calcined at 550 °C for 4 h in a muffle furnace. After cooling to RT, a yellow powder of gC_3_N_4_ nanosheets was obtained.

### 3.4. Synthesis of gC_3_N_4_-CoSe_2_ Nanocomposites

The gC_3_N_4_-CoSe_2_ nanocomposites were synthesized using a simple hydrothermal method. Firstly, 0.5 g of Co(CH_3_COO)_2_·4H_2_O, 0.8 g of Na_2_SeO_3_ and 6 mL of ethylenediamine were mixed with 50 mL of DI water and stirred for 10 min. Next, carbohydrazide (40 mmol) was slowly added dropwise to the solution and continuously stirred for 60 min. Subsequently, 0.3 g of gC_3_N_4_ nanosheets was introduced into the CoSe_2_ precursor solution and stirred for an additional 30 min. The final mixture was then transferred into a 100 mL Teflon-lined autoclave and heated at 180 °C for 18 h. After cooling to RT, the colloidal gC_3_N_4_-CoSe_2_ wet powder was collected, cleaned three times with DI water, and dried at 80 °C for 12 h to attain the gC_3_N_4_-CoSe_2_ nanocomposites.

### 3.5. Material Characterization

The microstructure and the elemental composition of the CoSe_2_ and gC_3_N_4_-CoSe_2_ catalysts were analyzed by using scanning electron microscopy (FE-SEM, Clara LMH, Tescan, Brno, Czech Republic) equipped with an in-situ energy-dispersive X-ray spectroscopy (EDX), respectively. For SEM-EDX analysis, a small amount of the sample was placed onto a double-sided carbon tape and gently air-blown to remove loosely bound excess particles prior to measurement. The structural properties of the catalysts were inspected by X-ray diffraction (XRD, D8-Advance system, Bruker, Billerica, MA, USA) measurement by using Cu Kα radiation (λ = 1.5406 Å), with the dried powders uniformly spread on the sample holders. The ionic states of the bare CoSe_2_, pristine gC_3_N_4_, and gC_3_N_4_-CoSe_2_ nanocomposites were examined employing X-ray photoelectron spectroscopy (XPS, ESCALab250Xi system, Thermos Fisher Scientific, Waltham, MA, USA) using vacuum-dried samples mounted on carbon tape. The textural properties of the catalysts were evaluated using Brunauer–Emmett–Teller (BET, BELSORP-mini II system, MicrotracBEL, Osaka, Japan) and Barrett–Joyner–Halenda (BJH) analyses after degassing the samples under vacuum prior to nitrogen adsorption measurements.

### 3.6. Electrocatalytic HER Measurements

The electrocatalytic HER performance of the CoSe_2_ nanoparticles and gC_3_N_4_-CoSe_2_ nanocomposites were evaluated employing a VersaSTAT3 electrochemical analyzer (Ametek Scientific Company, Mahwah, NJ, USA) in a three-electrode setup with 1 M KOH. To prepare the working electrodes, the catalysts (i.e., CoSe_2_ nanoparticles and gC_3_N_4_-CoSe_2_ nanocomposites) were dispersed in N-methyl-2-pyrrolidone and coated onto nickel foam (NF, 1 × 1 cm) substrates, followed by drying at 80 °C for 8 h using an air-circulating electric oven. A coiled platinum wire served as the counter electrode, while a saturated calomel electrode (SCE) was used as the reference electrode. The electrocatalytic measurements including cyclic voltammetry (CV), linear sweep voltammetry (LSV), chronopotentiometry (CP), and electrochemical impedance spectroscopy (EIS) were performed to assess the electrochemical performance and the stability of the prepared catalysts. The CV tests were performed in the potential window of 0 to 0.5 V with scan rates between 10 and 100 mV/s. The LSV measurements were investigated at −1 to −1.8 V under a persistent scan rate of 1 mV/s. Additionally, the CP characteristics were assessed at various current densities (i.e., −10 to −100 mA/cm^2^), with each step maintained for 10 min. The EIS test was performed over a frequency domain of 1 Hz to 10 kHz with an AC amplitude of 10 mV. The double-layer capacitance (*C*_DL_) was obtained from the non-faradaic CV region and used to calculate the electrochemically active surface area (ECSA) using the following equations [65]:

(1) $$J_{\mathrm{DL}}=C_{\mathrm{DL}}\;\times \nu /A$$

(2) $$ECSA=C_{DL}/C_{e}$$

where *C*_DL_, *J*_DL_, *C*_e_, *A*, and *v* are the double-layer capacitance, non-Faradaic charging current, KOH specific capacitance (KOH ~ 0.04 mF/cm^2^), fabricated electrode area, and the scan rate, respectively. The overpotential (*η*) and Tafel slope (*S*_T_) of HER was calculated using from the below equations [66,67,68,69,70,71,72]:

(3) $$E_{\mathrm{RHE}}=E_{\mathrm{SCE}}+{E^{0}}_{\mathrm{SCE}}+0.059\mathrm{pH}$$

(4) $$\eta =E_{\mathrm{RHE}}$$

(5) $$\eta =S_{T}\log\;\left(J\right)+c\;$$

where *E*^0^_SCE_, *η*, *J*, *c*, and *E*_RHE_ are the SCE potential, overpotential, current density, fitting parameter, and the reversible hydrogen electrode potential.

## 4. Conclusions

The high-performance HER electrocatalyst of gC_3_N_4_-CoSe_2_ nanocomposites was successfully synthesized using a simple hydrothermal approach. The gC_3_N_4_-CoSe_2_ nanocomposites showed an interconnected and aggregated morphology with a high mesoporous structure. The gC_3_N_4_-CoSe_2_ catalyst delivered a lower *η* of 141 mV and small *S*_T_ of 62 mV/dec, demonstrating its excellent intrinsic HER activity. Moreover, the gC_3_N_4_-CoSe_2_ nanocomposites displayed an increased number of active sites and lowered interfacial resistance facilitated faster charge transfer and efficient ion transport. The long-term CP stability test conducted for 100 h at 10 mA/cm^2^ endorsed the catalysts mechanical robustness and outstanding durability. These results suggest that the hydrothermally synthesized gC_3_N_4_-CoSe_2_ nanocomposites are promising materials for efficient electrocatalytic water splitting.

## Figures and Tables

**Table 1:** Comparison of HER performance for CoSe_2_ and gC_3_N_4_-CoSe_2_ with previously reported electrocatalysts.

Catalyst	Current Density (mA/cm^2^)	Overpotential η_10_ (mV)	Tafel Slope (mV/dec)	Electrolyte	Reference
gC_3_N_4_-CoSe_2_	10	141	62	1 M KOH	This work
CoSe_2_	10	187	76	1 M KOH	This work
Three-dimensional CoSe_2_/CFF	10	141	68	0.5 M H_2_SO_4_	[53]
g-C_3_N_4_-MoS_2_	10	156	101	1 M KOH	[33]
CoSe_2_/MoO_2_/MoSe_2_	10	230	36.8	0.5 M H_2_SO_4_	[48]
MoS_2_/g-C_3_N_4_	10	240	63	1 M KOH	[54]
CoSe_2_-gC_3_N_4_/NF	50	210	84	1 M KOH	[55]
CoSe_2_-g-C_3_N_4_/GCE	20	193	-	0.5 M H_2_SO_4_	[28]
MoS_2_/NiSe_2_/rGO	10	127	73	1 M KOH	[56]
MoO_3_/AC	10	353	124	1 M KOH	[57]
S-gC_3_N_4_/NiV LDH	10	560	79	1 M KOH	[58]
CoSe_2_/C	10	189	34	1 M KOH	[40]
CoSe_2_/CNT	10	180	35	0.5 M H_2_SO_4_	[59]
CoSe_2_/NC-170	10	159	83	0.5 M H_2_SO_4_	[60]
MoS_2_/WS_2_ NF	10	251	61	1 M KOH	[61]
CoSe_2_/MoSe_2_	10	218	76	1 M KOH	[62]
CoSe_2_|CoP/CFP	10	140	42	0.5 M H_2_SO_4_	[63]

## Data Availability

The original contributions presented in this study are included in the article/Supplementary Materials. Further inquiries can be directed to the corresponding authors.

## Supplementary Materials

The following supporting information can be downloaded at: https://www.mdpi.com/article/10.3390/ijms262412188/s1. Refs. [20,33,34,52,54,58,61,73,74,75,76,77,78,79,80] are cited in Supplementary Materials file.
