# Supplementary material for: Graphitic Carbon Nitride-Decorated Cobalt Diselenide Composites for Highly Efficient Hydrogen Evolution Reaction
Source: Int J Mol Sci. 2025 Dec 18;26(24):12188. doi: 10.3390/ijms262412188 (PMC12733567; doi:10.3390/ijms262412188)
Supplement: Supplementary file 1 [file ijms-26-12188-s001.zip › ijms-4006383-supplementary.pdf]

Supplementary Data

Graphitic Carbon Nitride-Decorated Cobalt Diselenide Composites for Highly Efficient Hydrogen Evolution Reaction

Abu Talha Aqueel Ahmed [1], Saravanan Sekar [2], Sutha Sadhasivam [3], Balaji Murugan [4], Sangeun Cho [1], Youngmin Lee [1,5], Sejoon Lee [1,5] and Sankar Sekar [1,5,*]

[1] Division of System Semiconductor, Dongguk University-Seoul, Seoul 04620, Republic of Korea
[2] Department of Mechanical Engineering, K. Ramakrishnan College of Technology, Trichy 621112, Tamil Nadu, India
[3] Department of Chemistry, CMS College of Engineering, Namakkal 637003, Tamil Nadu, India
[4] Department of Electrical & Computer Engineering, National University of Singapore, Singapore 117608, Singapore
[5] Quantum-Functional Semiconductor Research Center, Dongguk University-Seoul, Seoul 04620, Republic of Korea
* Correspondence: sanssekar@dongguk.edu

■ Surface Characteristics of $CoSe_2$, gC_3N_4 and gC_3N_4-$CoSe_2$

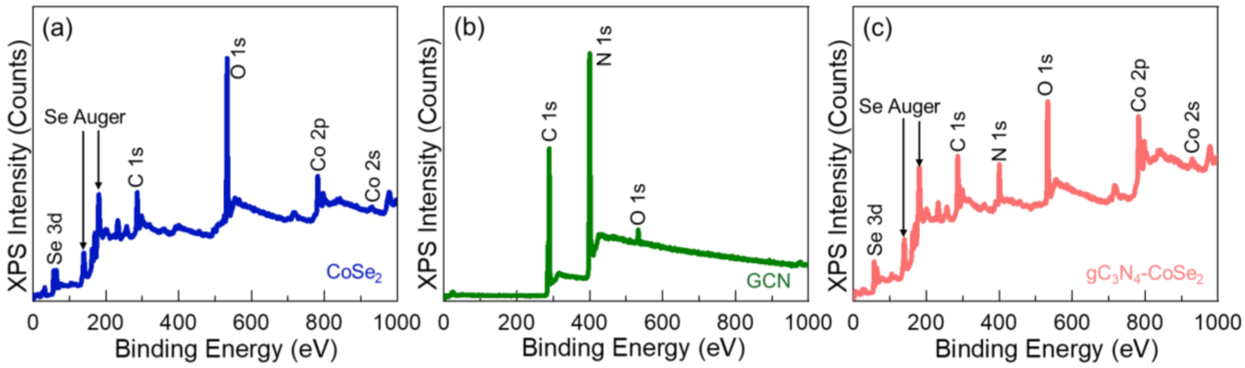

Figure S1: Full survey XPS spectra of the $CoSe_2$, gC_3N_4 and gC_3N_4-$CoSe_2$ nanocomposites.

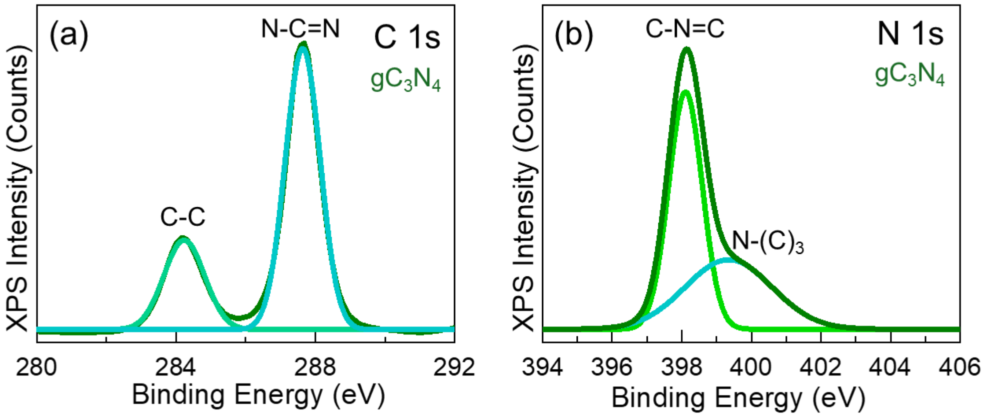

Figure S2: (a) C 1s and (b) N 1s of the core level XPS spectra of the gC_3N_4 nanosheets.

■ Electrochemical Properties of $CoSe_2$ and gC_3N_4-$CoSe_2$

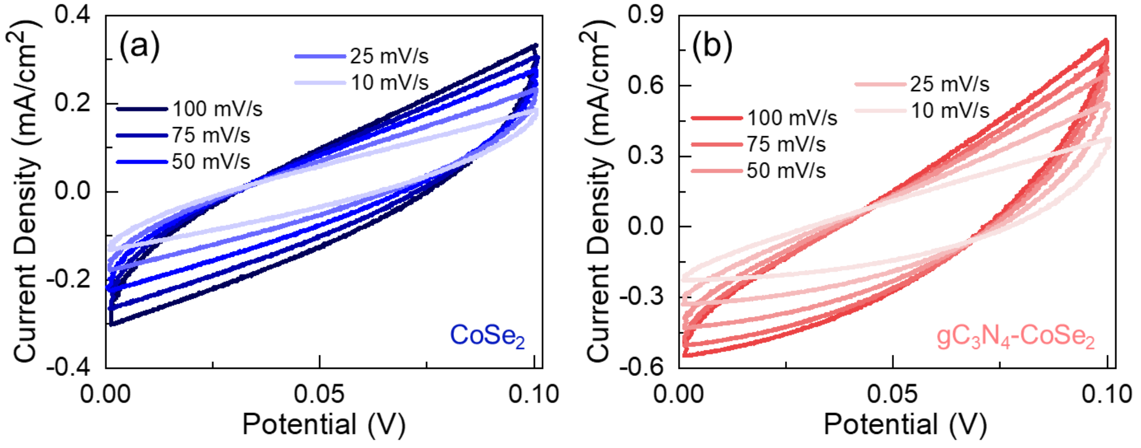

Figure S3: Non-faradic CV curves of (a) $CoSe_2$ and (b) gC_3N_4-$CoSe_2$ catalysts.

■ Electrocatalytic Properties of $CoSe_2$ and gC_3N_4-$CoSe_2$ before and after Stability Test

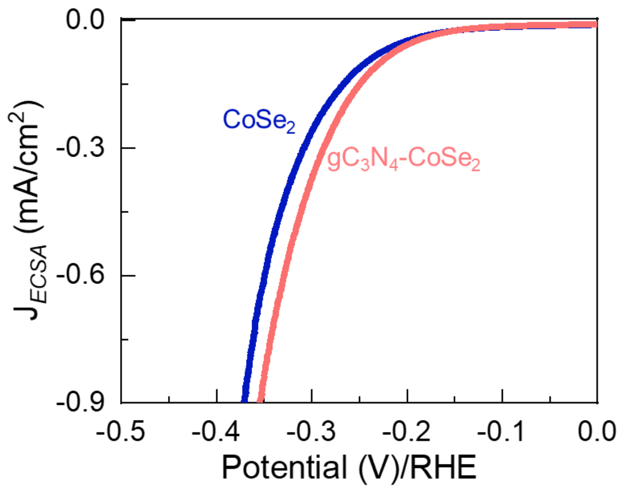

Figure S4: ECSA normalized LSV curves of the $CoSe_2$ and gC_3N_4-$CoSe_2$ catalysts.

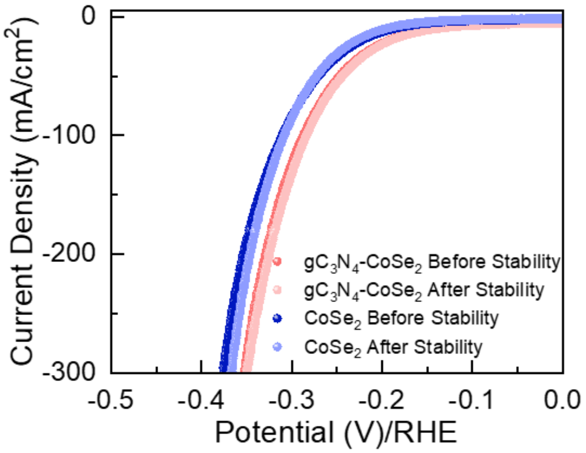

Figure S5: LSV curves of $CoSe_2$ and gC_3N_4-$CoSe_2$ before and after HER stability test.

■ Morphological Properties of $CoSe_2$ and gC_3N_4-$CoSe_2$ After Stability Test

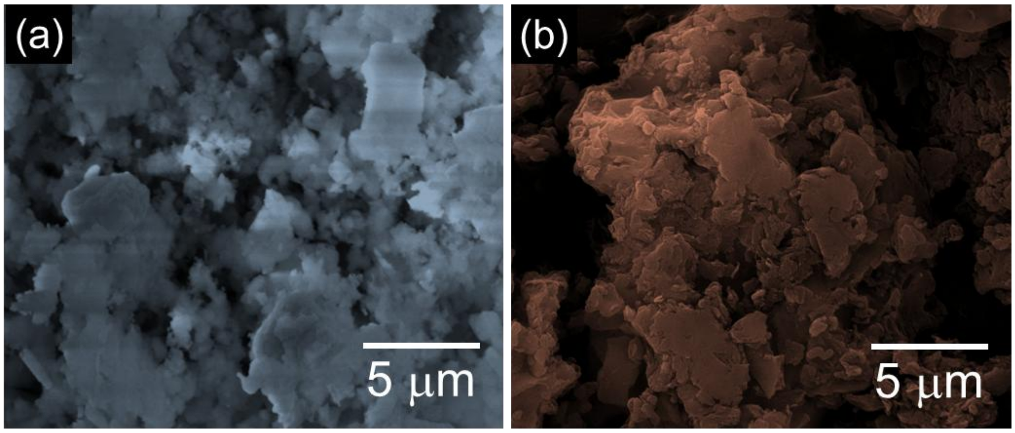

Figure S6: FE-SEM images of the (a) $CoSe_2$ and the (b) gC_3N_4-$CoSe_2$ catalysts after the stability test.

Table S1 Comparison of BET specific surface area for $CoSe_2$ and gC_3N_4-$CoSe_2$ with previously reported electrocatalysts.

Catalyst	Specific Surface Area (m^2/g)	Reference
gC_3N_4-$CoSe_2$	**82**	**This work**
$CoSe_2$	**35**	**This work**
$CoSe_2$ Rods	27.5	[20]
g-C_3N_4-MoS_2	46	[33]
$CoSe_2$	44.3	[34]
$CoSe_2$/ gC_3N_4	75.4	[34]
MoS_2/g-C_3N_4	16.26	[54]
S-gC_3N_4/NiV LDH	64.7	[58]
MoS_2/WS_2 NF	3.72	[61]
$CoSe_2$	11.6	[73]
$CoSe_2$	21.24	[74]
$CoSe_2$-NC@HCF	70.4	[75]
$CoSe_2$/CdS	13.03	[76]
$CoSe_2$/$Ti_3C_2T_x$	29.15	[77]
$CoSe_2$/NC	11.77	[78]
3-$CoSe_2$/g-C_3N_4	31.98	[52]
25 wt.% g-C_3N_4/ZnO	15.45	[79]
g-C_3N_4-NS/$ZnMoO_4$	32.4	[80]

References

20. Lan, K.; Li, J.; Zhu, Y.; Gong, L.; Li, F.; Jiang, P.; Niu, F.; Li, R. Morphology engineering of $CoSe_2$ as efficient electrocatalyst for water splitting. J. Colloid Interface Sci. 2019, 539, 646-653, doi:https://doi.org/10.1016/j.jcis.2018.12.044.

33. Sekar, S.; Shanmugam, A.; Lee, Y.; Lee, S. Highly Efficient Electrocatalyst of 2D–2D gC3N4–MoS2 Composites for Enhanced Overall Water Electrolysis. Materials 2025, 18, 3775.

34. Wang, R.; Dai, A.; Vijayalakshmi, M.; Jang, W.Y.; Kakarla, R.R.; Shim, J.; Aminabhavi, T.M.; Reddy, C.V. Synthesis of novel 2D g-C_3N_4/3D $CoSe_2$ hierarchical microflower-like hybrids for high-performance energy-storage applications. J. Energy Storage 2024, 104, 114577, doi:https://doi.org/10.1016/j.est.2024.114577.

52. Hao, Q.; Liu, Y.; Zou, R.; Shi, G.; Yang, S.; Zhong, L.; Yang, W.; Chi, X.; Liu, Y.; Admassie, S.; et al. g-C_3N_4 nanosheets coupled with $CoSe_2$ as co-catalyst for efficient photooxidation of xylose to xylonic acid. Green Energy Environ. 2025, 10, 231-238, doi:https://doi.org/10.1016/j.gee.2024.04.004.

54. Fageria, P.; Sudharshan, K.Y.; Nazir, R.; Basu, M.; Pande, S. Decoration of MoS_2 on g-C_3N_4 surface for efficient hydrogen evolution reaction. Electrochim. Acta 2017, 258, 1273-1283, doi:https://doi.org/10.1016/j.electacta.2017.11.184.

58. Srividhya, G.; Viswanathan, C.; Ponpandian, N. Interfacing NiV layered double hydroxide with sulphur-doped g-C_3N_4 as a novel electrocatalyst for enhanced hydrogen evolution reaction through Volmer–Heyrovský mechanism. Energy Adv. 2023, 2, 1464-1475, doi:10.1039/D3YA00287J.

61. Nguyen, T.V.; Tekalgne, M.; Tran, C.V.; Nguyen, T.P.; Dao, V.; Le, Q.V.; Ahn, S.H.; Kim, S.Y. Synthesis of MoS_2/WS_2 Nanoflower Heterostructures for Hydrogen Evolution Reaction. Int. J. Energy Res. 2024, 2024, 3192642, doi:https://doi.org/10.1155/2024/3192642.

73. Li, W.; Zheng, M.; Tian, Z.; Long, G.; Zhang, S.; Chen, Q.; Zhong, Q. Coral-Like $CoSe_2$-Nitrogen-Doped Porous Carbon as Efficient Counter Electrodes for Quantum Dot Sensitized Solar Cells. ECS J. Solid State Sci. Technol. 2021, 10, 045012, doi:10.1149/2162-8777/abe97d.

74. Joseph, A.; Kumar, D.S.H.; Ramadoss, M.; Muralidharan, K. Organic-free $NiSe_2$, $CoSe_2$, and $NiSe_2$/$CoSe_2$ for non-enzymatic glucose sensing. J. Solid State Electrochem. 2025, 29, 4461-4471, doi:10.1007/s10008-025-06310-4.

75. Xinxing, S.; Hongjing, G.; Shuangke, L.; Weiwei, S.; Yujie, L.; Danqin, W.; Qingpeng, G.; Xiaobin, H.; Jing, X.; Chunman, Z. Space-confined synthesis of $CoSe_2$-NC nanoclusters anchored on honeycomb-like carbon framework towards high-performance lithium sulfur battery. Ionics 2023, 29, 4707-4722, doi:10.1007/s11581-023-05164-y.

76. Gan, R.; Ma, X.; Wang, G.; Jin, Z. $CoSe_2$ Clusters as Efficient Co-Catalyst Modified CdS Nanorod for Enhance Visible Light Photocatalytic H2 Evolution. Catalysts 2019, 9, 616.

77. Yan, Z.; Li, J.; Chen, Q.; Chen, S.; Luo, L.; Chen, Y. Synthesis of $CoSe_2$/MXene composites using as high-performance anode materials for lithium-ion batteries. Adv. Compos. Hybrid Mater. 2022, 5, 2977-2987, doi:10.1007/s42114-022-00524-0.

78. Yu, B.; Tressel, J.; Cui, T.; Pan, D.; DuBois, D.B.; Jones, C.; Hou, B.; Mayford, K.; Liu, Q.; Bridges, F.; et al. Rapid synthesis of carbon-supported CoS_2/$CoSe_2$ heterostructures by magnetic induction heating for efficient hydrogen

evolution reaction in acidic media. J. Power Sources 2025, 641, 236897, doi:https://doi.org/10.1016/j.jpowsour.2025.236897.
79. Wang, Y.; Liu, Z.; Li, Y.; Yang, X.; Zhao, L.; Peng, J. Boosting Photocatalytic Performance of ZnO Nanowires via Building Heterojunction with g-C_3N_4. Molecules 2023, 28, 5563.
80. Mousavi, M.; Moradian, S.; Pourhakkak, P.; Zhang, G.; Habibi, M.M.; Madadi, M.; Ghasemi, J.B. Fabrication of S-scheme heterojunction g-C_3N_4-nanosheet/$ZnMoO_4$ nanocomposite with high efficiency in photocatalytic N_2 fixation and Cr(VI) detoxification. J. Mater Sci. 2022, 57, 9145-9163, doi:10.1007/s10853-022-07225-5.
